# Current Challenges and Future Promise for Use of Extracellular Matrix Scaffold to Achieve the Whole Organ Tissue Engineering Moonshot

**DOI:** 10.1093/stcltm/szad046

**Published:** 2023-08-17

**Authors:** Likitha Somasekhar, Leigh G Griffiths

**Affiliations:** Department of Cardiovascular Medicine, Mayo Clinic, Rochester, MN, USA; Department of Cardiovascular Medicine, Mayo Clinic, Rochester, MN, USA

**Keywords:** tissue engineering, extracellular matrix, scaffold, tissue engineered, vascular

## Abstract

Whole organ tissue engineering encompasses a variety of approaches, including 3D printed tissues, cell-based self-assembly, and cellular incorporation into synthetic or xenogeneic extracellular matrix (ECM) scaffolds. This review article addresses the importance of whole organ tissue engineering for various solid organ applications, focusing on the use of extracellular (ECM) matrix scaffolds in such engineering endeavors. In this work, we focus on the emerging barriers to translation of ECM scaffold-based tissue-engineered organs and highlight potential solutions to overcome the primary challenges in the field. The 3 main factors that are essential for developing ECM scaffold-based whole organs are (1) recapitulation of a functional vascular tree, (2) delivery and orientation of cells into parenchymal void spaces left vacant in the scaffold during the antigen elimination and associated cellular removal processes, and (3) driving differentiation of delivered cells toward the appropriate site-specific lineage. The insights discussed in this review will allow the potential of allogeneic or xenogeneic ECM scaffolds to be fully maximized for future whole organ tissue-engineering efforts.

## Graphical Abstract

**Graphical Abstract:**
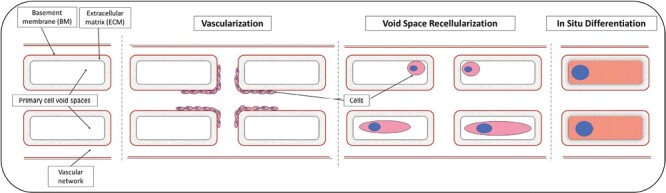

**Significance Statement**

This review addresses the importance of whole organ tissue engineering for various solid-organ applications, focusing on the use of extracellular (ECM) matrix scaffolds in such engineering endeavors. In this work, we focus on the emerging barriers to translation of ECM scaffold-based tissue engineered organs and highlight potential solutions to overcome the primary challenges in the field. The insights discussed in this review will allow the potential of allogeneic or xenogeneic ECM scaffolds to be fully maximized for future whole organ tissue engineering projects.

## Introduction

Currently, approximately 110 000 patients are on the US organ transplant waiting list.^1-3^ Donor organ shortage leads to ~15 waiting list patient deaths per day.^2^ While the short-term success rate of organ transplants has dramatically increased over time, complications related to immune rejection and the use of immunosuppressive drugs continue to limit long-term patient and/or graft survival.^4-6^ Whole organ tissue engineering aims to address the lack of organ availability by in vitro generation of viable human organs. Additionally, the use of autologous patient cells has the potential to overcome complications associated with rejection and use of immunosuppressive drugs in transplant recipients. Consequently, “off the shelf” tissue-engineered organs provide an enticing potential solution to overcome the limitations of allograft organ transplantation, by providing essentially unlimited organ supply while avoiding immune-mediated complications.

Whole organ tissue engineering encompasses a variety of approaches, including 3D printed tissues, cell-based self-assembly, and cellular incorporation into synthetic, allogeneic, or xenogeneic extracellular matrix (ECM) scaffolds. Recapitulating the native structure, composition, and function of native tissue extracellular environment is challenging with synthetic, printing, or self-assembly-based tissue engineering approaches. By comparison, allogeneic or xenogeneic ECM scaffolds provide a potentially significant advantage in that they inherently possess the complexity of native tissue ECM structure/composition/function properties. The degree to which native tissue complexity is preserved varies depending on the specific antigen elimination/decellularization scaffold generation approach used.^7-12^ Ongoing research continues to expand our knowledge of production methods, success criteria, and biological functions of unfixed ECM scaffolds toward increasing the scope and success of their clinical application. Acellular unfixed ECM scaffolds have attained widespread clinical use, with variable results depending on the application site, specific biomaterial, and processing method used.^13-15^ In cases where native ECM properties are retained, both allogeneic and xenogeneic ECM scaffolds have been shown to influence cell behavior, directing repopulating cell differentiation, proliferation, migration, phenotype, and function.^7,16,17^ This review focuses on the challenges associated with the use of xenogeneic ECM scaffolds in whole organ tissue engineering applications.

The primary goal in generation of acellular ECM scaffolds is to eliminate antigenic tissue components while retaining the native tissue ECM structure, composition, mechanical properties, and biological function.^18^ Recent work has made significant progress in defining specific antigenic barriers to xenotransplantation, exploring extraction conditions necessary to eliminate such antigens from candidate xenogeneic tissues, and establishing levels of antigen elimination required to achieve recipient graft-specific adaptive immune avoidance.^11,19-21^ Similarly, retention of native ECM structure, composition, and functional properties have all been shown to be instrumental in avoiding pro-inflammatory, while promoting pro-regenerative innate immune responses.^11,12^ The immunology of allogeneic or xenogeneic ECM scaffolds and strategies to overcome this challenge have been reviewed in detail elsewhere.^7,8,18,22^ As the challenge of producing immunologically acceptable acellular ECM scaffolds draws near accomplishment, research has increasingly shifted toward their utilization in tissue engineering applications. This review will focus on the emerging barriers to translation of ECM scaffold-based tissue-engineered organs and highlight potential solutions to overcome the primary challenges in the field. The primary barriers to the development of ECM scaffold-based whole organs are (1) Recapitulation of the entire vascular tree, which is essential to maintain the function of cells within the engineered organ and to allow communication with the central circulatory system. (2) Delivery and orientation of cells into the parenchymal void spaces left vacant in the scaffold during the antigen elimination and associated cellular removal processes. (3) Driving differentiation of delivered cells toward the appropriate site-specific lineage. Addressing these 3 primary obstacles will allow the potential of xenogeneic ECM scaffolds to be fully maximized for whole organ tissue engineering.

## Recapitulation of the Vascular Tree

The provision of vasculature is essential for whole organ tissue engineering to support cellular metabolic demands. This requirement stems from limitations of diffusion to sustain cell survival in engineered tissues.^23^ In avascular constructs, as tissue thickness increases, nutrient and waste elimination requirements in the center of the graft outstrip diffusional supply, resulting in core necrosis.^24^ Although novel methods for overcoming diffusional distance limitations have been investigated in vitro (eg, microporous constructs), important barriers remain to their use in whole organ engineering.^25,26^ First, although microporous approaches can facilitate in vitro diffusion, fabrication of an appropriately organized hierarchical vascular tree to facilitate anastomosis to a recipient’s vasculature in vivo remains challenging. Furthermore, both hierarchy of the vascular tree and functional architecture of the native vascular wall are essential for regulation of organ perfusion, predominantly via arteriolar vasoactive responses.^27^ Finally, native vasculature also serves a critical role in regulating tissue hemostasis via endothelial cell (EC) and ECM (eg, tissue factor) dependent mechanisms.^28^ Consequently, harnessing the native vascular structure, composition, and hierarchical organization of xenogeneic ECM scaffolds has potential to provide an ideal substrate for in vitro or in vivo whole organ perfusion, overcoming diffusional limitations to cell survival.

### ECM Scaffold Generation: Eliminating Antigens While Avoiding Off-Target ECM Damage

A wide range of decellularization or antigen removal approaches have been investigated and determined to be variably successful in generating acellular ECM scaffolds from candidate tissues and organs. Decellularization protocols utilizing detergents (eg, sodium dodecyl sulfate (SDS), sodium deoxycholate (SDC), and Triton-X 100), enzymatic digestion (eg, Trypsin), and/or osmotic lysis (eg, hypotonic solutions) have been shown to effectively remove cells from a variety of tissues.^29-32^ However, the effectiveness of varying approaches on maintaining native ECM structure-function properties depends on the particular chemical regime used. Such protocols have been applied via either diffusion (eg, small tissue pieces) or perfusion (ie, whole organ vascular perfusion) based protocols.^33,34^

Treatment of whole organs with perfusion-based decellularization has had varying effects on the resultant ECM and associated vascular structures.^35,36^ The effect of various decellularization or antigen removal methods on resultant ECM structure is to some extent predictable, considering the mechanism of action of the compounds used during scaffold generation. The most commonly used decellularization compound (ie, SDS) for example is a denaturing detergent. The denaturing effect of SDS combined with its high protein binding affinity result in the disruption of integral ECM proteins.^37,38^ SDS has been shown to intercalate in the triple helical structure of collagen, resulting in perturbation of collagen macromolecular structure, reducing scaffold collagen content, disrupting the elastin network, and altering resultant ECM scaffold functional properties.^37,39-41^ Use of the non-ionic detergent Triton X did not affect the structural integrity of the ECM components such as elastin and collagen II,^29^ but compromised collagen III content and degraded collagen IV integrity of the ECM. Decellularization of the scaffold using osmotic lysis was effective in removing endothelial cells, but ineffective in removing interstitial cellular elements.^42^ Furthermore, experience with SynerGraft heart valves suggests that hypotonic lysis alone results in inadequate elimination of antigenic components to overcome in vivo graft-specific adaptive immune responses, with severe adaptive immune destruction of the valve biomaterial reported in human patients.^43^ Both non-specific (eg, trypsin) and targeted (eg, alpha-galactosidase) enzyme-based protocols have been used in decellularization protocols to reduce biomaterial antigen content.^44,45^ As might be expected, off-target disruption of structural ECM proteins with non-specific enzymatic approaches has generally been reported to have more detrimental effects on the resultant ECM than that seen with targeted enzymatic approaches.^44,45^ For instance, non-specific enzymatic digestion led to distortion and fragmentation of elastic fibers present on the ECM, resulting in altered mechanical behavior and reduced extensibility by increasing the scaffold stiffness.^46^ Conversely, targeted enzymatic antigen elimination approaches (eg, alpha-galactosidase) are effective in reducing individual antigen content and avoiding off-target damage to the remaining ECM.^44^ However, targeted enzymatic approaches are frequently combined with other approaches, since they fail to eliminate cellular or antigenic components that are not associated with the primary enzymatic target. For instance, Wu et al optimized SDS decellularization of an acellular porcine annulus fibrosus scaffold by including α-Gal epitope reduction (ie, targeted antigen removal) using alpha-galactosidase administration.^47,48^ Consequently, consideration of the mechanism of action of chemicals used in decellularization/antigen removal approaches and their effect on the resultant biomaterial are critical considerations in ECM scaffold generation.

### Preserving the Native ECM Vascular Tree

**Figure 1.**
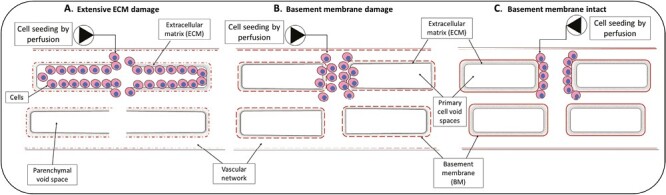
Location of seeded endothelial cells following perfusion-based seeding is dependent on integrity of the vascular tree basement membranes. **A**. Cells repopulating into the parenchymal void spaces due to extensive basement membrane damage. **B**. Cells repopulating the surface and may penetrate the void space region due to partial BM damage. **C**. Cells repopulating on the vascular tree of the intact ECM when the vascular basement membrane is intact. Figure represents cardiac muscle with one capillary per cardiomyocyte (ie, parenchymal void space).

The challenge of preserving native ECM properties following the ECM generation process is magnified when considering the delicate architecture, composition, and resultant function of native vascular tissue networks and particularly capillary beds. While large-scale vascular structures are generally well preserved (eg, coronary arteries and their proximal branches), distal vessels in the vascular tree of solid organs are largely disrupted (eg, capillaries) by reported decellularization approaches.^35,36^ Some groups have demonstrated intact vascular structures and successful perfusion recellularization of vessels as small as 10 µm.^33,35,49^ However, cellular repopulation or transit across capillary beds to the venous vasculature was reported to be infrequent, likely due to disruption of at least some portions of the capillary bed during the decellularization process.^35^ Even in instances where the capillary-like structures were identified following perfusion seeding of rabbit whole heart scaffolds with human induced pluripotent stem-cell derived endothelial cells, the number of repopulated capillary-like structures was extremely small compared to the expected number for myocardial tissue (ie, approximately 1 capillary per cardiomyocyte).^49^ Similarly, successful dye transit across arterial to venous vascular beds has also been reported.^29,50,51^ While these results suggest the potential for intact capillary beds, it is also possible that leakage into the scaffold parenchyma may occur from smaller vessels, and then re-join the venous vascular tree at larger venules/veins, giving the impression of an intact vascular network (Fig. 1).^35^ Consequently, careful examination of the vascular tree, interstitial spaces, and organ parenchyma are all required to conclusively determine the circulatory pathway in cell-seeded or dye-perfused ECM scaffolds. Furthermore, simple presence of a tubular interconnected vascular network is insufficient to confirm retention of critical compositional and functional properties of ECM vascular structure. For instance, even in scaffolds that appear to conduct fluid, content, and organization of critical ECM proteins such as collagen and elastin are often disrupted relative to native tissue.^33,36,52^ The functional importance of such structural ECM macromolecules is well documented in large diameter vessels; where content and organization of collagen and elastin are critical for retention of native vascular tissue mechanical properties.^39^ Consequently, assessment of the content, structure, organization, and function of ECM macromolecules has important implications for vascular integrity and functional properties. Progress in achieving the goal of preservation of native vascular networks requires development of non-toxic whole organ ECM scaffold generation methods capable of retaining intact tubular interconnected arteries, arterioles, capillaries, venules, and veins, while simultaneously maintaining content, organization, macromolecular structure, and function of integral vascular ECM proteins.

The ubiquitous production of basement membrane (BM) components by vascular ECs provides a marker of vascular network integrity and important functional cues to guide repopulating cell behavior. The importance of individual BM components has been well established in hydrogel models in vitro and reviewed elsewhere.^53^ Recent studies utilizing ECM scaffolds have demonstrated the potential for BM components (eg, Col IV, Laminin) to modulate repopulating EC phenotype, function, migration, and monolayer formation.^54,55^ Critically, BM components have been reported to prevent Z-direction (ie, translocation of cells across the planar surface of the vessel luminal surface into the parenchymal void spaces left by primary cell removal) migration of seeded cells. In the case of ECs, the capacity of BMs to prevent Z-direction migration has potential to guide to facilitate cellular repopulation of the entire vascular network (ie, seeded ECs are confined to the vascular lumen by intact BM).^16,54^ However, the delicate structure of the BM has proven extremely challenging to maintain in decellularized ECM scaffolds. For instance, Faulk et al examined the effect of 4 different detergent-based decellularization methods (ie, SDS, Triton X-100, sodium deoxycholate, or CHAPS) on BM integrity.^34^ The authors demonstrated disruption of BM components (ie, Col IV, VII, and laminin) using all decellularization methods, although the extent of disruption varied with the detergent used. Triton X-100 and sodium deoxycholate treated scaffolds retained some staining for Col IV for instance, which may explain the compatibility of these scaffolds with human EC repopulation and monolayer formation, although laminin and Col VII disruption were extensive.^34^ Similarly, Schneider et al demonstrated disruption of placental artery BM integrity following either SDS or Triton X-100 decellularization.^56^ Alternatively, Shupe et al reported preservation of laminin in large diameter vessels of whole liver scaffolds formed using Triton X-100 followed by 0.1% SDS decellularization.^57^ However, capillary laminin staining and Col IV presence were incompletely preserved. Finally, Calle et al used a proteomic method to show that lung scaffolds decellularized with a “gentle” Triton X-100, sodium deoxycholate method preserved 75% of the analyzed BM proteins at near-native levels.^58^ As expected, preservation of BM components enhanced endothelial and mixed lung cell repopulation of the resultant scaffold following perfusion-based seeding. However, this “gentle” approach resulted in increased retention of cellular proteins compared to traditional CHAPS decellularization methods.^58^ Conversely, use of the non-denaturing non-ionic detergents (eg, aminosulfabetaine 14 (ASB-14) or ASB-16) in antigen removal methods has been reported to eliminate antigenic components while retaining native tissue BM components (eg, Laminin, Collagen IV).^54,59^ Importantly, such antigen removed scaffolds have been shown to modulate appropriate quiescent monolayer formation of repopulating ECs, mirroring the behavior identified in single component hydrogel studies.^55,59^ Consequently, ongoing refinements of whole organ ECM scaffold production methods have potential to leverage BM integrity combined with perfusion-based EC seeding toward recapitulation of the native vascular tree.

### Cellular Repopulation of the ECM Scaffold Vascular Tree

The native vascular compartment is comprised of EC, vascular smooth muscle cells (vSMC’s), and perivascular MSC’s (pericytes). While all cell types are required for healthy vascular function, the importance of a quiescent EC monolayer in modulating vascular tone, coagulation state, platelet adhesion, barrier function, and leukocyte adhesion has led the majority of publications to focus on EC repopulation of whole organ grafts. However, the importance of other cell types, particularly pericytes, in maintaining healthy EC function has recently been appreciated.^60^ Indeed, publications examining coculture of ECs and pericyte precursors in the vascular compartment of whole organ ECM scaffolds have shown improvement in the extent of endothelialization compared to previously reported monoculture systems (Table 1A).^61,62^ A commonly pursued re-endothelialization approach utilizes human umbilical vein endothelial cells (HUVECs) as a source for EC’s, and is often co-seeded with human mesenchymal stem cells (hMSC) to serve as a perivascular cell population.^61^ Perivascular support from hMSCs was shown by Ren et al to improve EC monolayer coverage compared to EC seeding alone (54% vs. 33% coverage, respectively).^61^ Furthermore, other experiments have shown that co-seeding other cell types alongside endothelial cells can improve cellular function.^62^ Finally, continuous perfusion with seeded endothelial or endothelial progenitor cells has also been able to regenerate a relatively evenly distributed endothelium throughout whole organs.^62-64^ Consequently, although this work suggests that coculture and continuous perfusion may improve vascular endothelialization, future work focused on the proportion, sequence of delivery, duration of seeding, and culture conditions required to optimize the outcome for each cell type will be required to further improve vascular function in ECM scaffold-based tissue engineered constructs.

**Table 1.** Examples of (A) EC and pericyte precursor seeding strategies for repopulation of the vascular compartment of whole organ ECM scaffolds, (B) cell combinations and seeding strategies used for void space repopulation, (C) cellular differentiation of different cell combinations and culture strategies for whole organ recellularization.

(A) Recapitulation of vascular tree			
Scaffold type	Cell type	Culture conditions	Outcome
Pre-vascularized pancreatic decellularized scaffold.^65^	Endothelial progenitor cells (EPCs).	Endothelialization with endothelial progenitor cells (EPCs) was conducted in a bioreactor and was transplanted into the rat in vivo to observe the anastomosis.	EPCs can be located around the blood vessel wall, and re-endothelialized scaffold connected with the host through new blood vessel formation.
Decellularized rat heart.^35^	Rat aortic endothelial cells (RAECs).	Three strategies to recellularize perfusion-decellularized rat heart vasculature with RAECs: retrograde aortic infusion, brachiocephalic artery (BA) infusion, or a combination of inferior vena cava (IVC) plus BA infusion. The re-endothelialized scaffolds were maintained under vascular flow in vitro for 7 days.	Re-endothelialization of whole decellularized hearts throughout both arterial and venous beds and cavities by using arterial and venous delivery was achieved.
Decellularized porcinePericardium.^66^	Human adipose tissue-derived stem cells (ASCs) + HUVEC’s.	Static culture for 22 days.	Thin layers of human umbilical endothelial cells were formed within 4 days previously seeded with ASCs. ASCs are capable of accelerating in situ endothelialization.
Decellularized kidney scaffold.^67^	Human glomerular microvascular endothelial cells (hgMVECs) and human inducible pluripotent stem cell-derived endothelial cells (hiPSCs).	Decellularized rat kidney scaffolds were loaded with growth factors (eg, vascular epithelial growth factor (VEGF) and then perfusion recellularized via combined arterial and venous perfection using either hgMVECs and iPS-ECs.	Using a novel simultaneous arteriovenous delivery system, it was reported that re-endothelialization of the kidney vasculature, including the glomerular and peritubular capillaries was observed.
Decellularized human whole liver grafts.^68^	Umbilical vein endothelial endothelium cells.	HUVEC were incubated for 1-5 days with sections cut from DHLS (8 mm and 250 μm thick) in a 48-well plate in the HUVEC culture medium.	Scaffolds repopulated with HUVECs showed some tropism of reseeded cells to vasculature. However, some HUVECs also migrated to the parenchymal portions of the scaffold.
Decellularized whole mouse lungs.^69^	Pulmonary arterial endothelial cells (PAECs), microvascular endothelial cells (MVECs), and rat pulmonary microvascular endothelial progenitor cells (RMEPCs) were isolated from Sprague-Dawley rats and cultured.	Cultured for 8 days via pulsatile perfusion.	RMEPCs-reseeded in decellularized lung scaffolds displayed enhanced repopulation and functional barrier properties. PAECs and MVECs showed tropism for large or microscopic vessels respectively, while RMEPCs repopulated all vessel types.
Human liver scaffolds.^7^	Human parenchymal and non-parenchymal liver cell lines (HepG2 and LX2 cells), (HUVEC), primary human hepatocytes, and hepatic stellate cells.	Cultured for 7-14 days.	LX2 cells grown in 3D-scaffolds retained the phenotype of activated cells. Efficient re-endothelization obtained with human endothelial cells, although this was largely confined to large vessels.
(B) Cellular repopulation of void spaces			
Scaffold type	Cell type	Culture conditions	Outcome
Decellularized porcine Pericardium^66^	Human adipose tissue-derived stem cells (ASCs) + HUVEC’s	Static culture for 22 days.	ASCs grew only on the surface of the decellularized pericardium, the fibrin-modified scaffolds were entirely repopulated in 28 days, and the scaffolds modified with fibrin, heparin, and VEGF were already repopulated within 6 days.
Decellularized human lung and rat lungs^70^	iPSC-derived epithelial progenitor cells and rat microvascular endothelial cells (RLMEC)	Vascular perfusion using a bioreactor system and cultured for 4 days.	In the acellular human scaffolds, iPSC-derived progenitor cells repopulated the scaffold in both airways and the alveolar compartment. In the rat scaffold, progenitor cells derived from iPSC were distributed from airway to alveoli, and these engineered lungs displayed much of the microarchitecture of native rat lungs.
Rat decellularized kidney scaffolds^71^	Human adipose tissue-derived mesenchymal stem cells (AD-MSCs)	AD-MSCs were seeded onto the surface of SDS-treated scaffolds and assessed after 3 weeks of culture.	Cell attachment on the renal scaffold was observed after recellularization. Although only a small proportion of the seeded cells migrated into the scaffold (the rest remained on the surface).
Decellularized rat hearts^72^	Human embryonic stem cells (hESCs)-derived cardiovascular progenitor cells (CPCs)	Cultured for 12 days via perfusion.	Study revealed that tethered bFGF leads to superior results with higher cell attachment and retention into decellularized heart patches compared to passively absorbed bFGF or the absence of exogenously added growth factors. Observed spontaneous and synchronous contractions of humanized hearts as well as advanced alignment of myofilaments. However, percent repopulation of the parenchyma was between ~20% and 50% depending on cardiac location (ie, higher in LV free wall, lower in RV free wall and septum).
(C) Cell differentiation			
Scaffold type	Cell type	Culture conditions	Outcome
Rat decellularized kidney scaffolds^71^	Human adipose tissue-derived mesenchymal stem cells (AD-MSCs)	AD-MSCs were seeded onto the surface of SDS-treated scaffolds and assessed after 3 weeks of culture	The scaffolds did induce differentiation of these migrating cells toward epithelial and endothelial lineages
Perfusion skeletal muscle ECM (pM-ECM) was prepared from porcine rectus abdominis (RA)^73^	C2C12 (immortalized mouse myoblast cell line)	The pepsin-digested pM-ECM and SIS-ECM at concentrations of 50, 100, and 200 μg/ml of dry weight were added to cultured C2C12 cells	Digested pM-ECM drove myogenic differentiation of C2C12 cells, while SIS-ECM did not
Decellularized mouse heart scaffolds^74^	Mouse induced cardiac progenitor cells (iCPCs) reprogrammed from either lung or cardiac fibroblasts and labeled with CMV-GFP expressing lentivirus	Vascular perfusion—after 1 week, switched the perfusate to a differentiation medium (fibroblast medium) and maintained in culture for 3-5 weeks	The iCPCs readily attached to the decellularized scaffold and differentiated to relevant cardiac lineages including cardiomyocytes, endothelial, and smooth muscle cells. Cardiomyocytes in the heart scaffolds showed an increase in cell size and sarcomere organization compared to in vitro differentiated iCPCs over a similar period of time
Decellularized whole rabbit heart scaffolds^49^	Human-induced pluripotent stem cell-derived endothelial cells, cardiomyocytes, and other cardiac cell types	Cells were sequentially delivered to the scaffold using an optimized endothelial cell: cardiomyocyte media for 60 days	Macroscopic assessment after 60 days showed that the LV wall of recellularized hearts was anatomically restored to full thickness from base to apex and endocardium to epicardium. Vessel patency was demonstrated after perfusion of recellularized hearts transplanted into the femoral artery bed of a pig. However, capillary density was extremely low and parenchymal cellular repopulation was patchy
Decellularized rat hearts^72^	Human embryonic stem cells (hESCs)-derived cardiovascular progenitor cells (CPCs).	Cultured for 12 days via perfusion.	Immobilized bFGF to heart ECM—resulted in improved retention of CPCs and differentiation to cardiomyocytes, smooth muscle cells, and endothelial cells.
Human liver scaffolds^7^	Human parenchymal and non-parenchymal liver cell lines (HepG2 and LX2 cells), (HUVEC), primary human hepatocytes, and hepatic stellate cells	Cultured for 7-14 days	HepG2 cells grown in 3D scaffolds were able to maintain higher cellular differentiation features following prolonged culture when compared to the same cell preparation grown on standard 2D-plastic conditions

In addition to determining the optimal delivery of cell types to achieve construct revascularization, consideration must also be given to maintaining the health and function of these cells. Since almost all whole organ recellularization investigations are undertaken in vitro, the use of bioreactors to generate a specific set of conditions conducive to cell health is ubiquitously used. The effect of shear stress on endothelial development has been widely investigated, with custom-made bioreactors used to examine the effect of laminar versus pulsatile flow, both with mixed results.^75-78^ Some of these studies also investigated varying perfusion flow rates at different post-seeding times, suggesting that an increase of physiologic mechanical signals toward normal physiologic forces may drive complete monolayer coverage and endothelial cell maturation.^79-82^ This mimicking of physiologic conditions aims to gradually provide native mechanical stimuli and promote cellular function which approximates that seen in vivo. Overall, the current evidence supports the conclusion that a gradual increase in flow toward physiologic values results in enhanced cell adhesion, invasion, and proliferation. Future work is needed to determine the optimal timing, flow rate, modality (eg, pulsatile vs. laminar), and viscosity of perfusion to maximize in vitro and resultant in vivo tissue-engineered vascular health.

## Cellular Repopulation of Void Spaces

Recellularization of ECM scaffolds with parenchymal cells is essential to establish physiologic function of engineered tissues and organs. Physiologic activity of all tissues and organs is critically dependent on cellular composition, orientation, interactions (ie, cell-cell and cell-matrix), and function. To date, recapitulation of such sophisticated cellular architecture and activity has been hindered by our fundamental inability to achieve cellular repopulation of more than a fraction of the parenchymal void spaces created via primary cell removal during ECM scaffold production.^83-86^ Progress in cellular repopulation of ECM scaffolds must consider the route of cell seeding, guidance of cell migration within the scaffold, and the cell type/types with which to seed the scaffold to achieve maximal repopulation.

The decellularization process ideally removes all cellular material, leaving the extracellular matrix with parenchymal void spaces in which primary cells of the native tissue originally resided. A similar situation occurs in scaffolds fabricated through other methods; bio-printed matrices have organized spaces built in during the fabrication process, while hydrogel scaffolds sometimes use particle leaching to create a porous structure for instance.^87-89^ As previously discussed, acellular ECM scaffolds have already achieved clinical use in relatively planar applications, where short diffusion distances allow rapid in vivo cellular repopulation supported by later angiogenesis. Although such in vivo bioreactor approaches may be capable of restoring the partial function of larger organs (eg, skeletal muscle),^35,90-94^ implantations of acellular ECM scaffolds is unable to provide immediate functional benefit for critical organ defects (eg, myocardial infarction) or whole organ replacement (eg, heart, lung, or liver transplant) applications. Although in vivo cellular repopulation of parenchymal void spaces demonstrates the feasibility of generating functional tissue from ECM scaffolds, engineering whole organs with immediate post-implantation function requires in vitro recellularization. Further investigation of the mechanisms responsible for in vivo cellular parenchymal void space repopulation in ECM scaffolds is necessary to inform ongoing attempts to recapitulate such processes in vitro (Table 1B). Additionally, disruption of integral ECM protein macromolecular structure and residual toxicity of decellularization compounds have both been shown to negatively impact repopulating cell viability and function.^39,54^ Consequently, ongoing efforts to control in vitro void space repopulation of ECM scaffold are critically dependent on characterizing the extent to which differing scaffold generation methods preserve native ECM structure, function, composition relationships, and leveraging insights from in vivo cellular repopulation studies to enhance understanding of how to control in vitro cellular repopulation and function.

**Figure 2.**
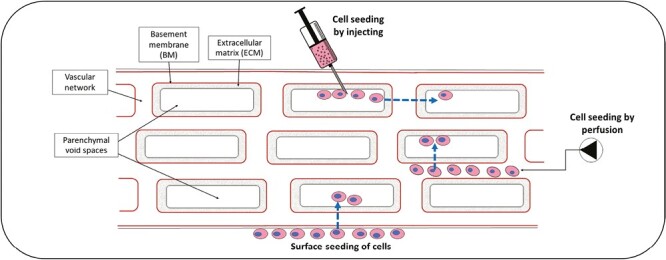
Different processes of cell seeding on scaffolds. Cells are seeded by injecting into the void space of the primary cell, by perfusion of the organ vasculature using a pump or by seeding on the surface of the peer review scaffold. Grey rectangle = cells, red lines = vascular basement membranes, blue-dotted arrow = migration required for seeded cells to enter parenchymal void spaces.

Cell seeding route plays an important role in in vitro cellular repopulation of ECM scaffold. Several studies have demonstrated the effect of local ECM niche on seeded cell behavior, which must therefore be considered in the chosen seeding route.^16,54,55^ Three predominant seeding strategies have been reported: (1) surface seeding, (2) intra-scaffold injection seeding, and (3) perfusion seeding (Fig. 2).^91,95-97^ Each of these approaches expose cells to differing ECM niche which exert potentially predictable effects on resultanT-cell behavior.

### Seeding Cells on the ECM Scaffold Surface

Due to its simplicity, surface seeding has been used for the majority of ECM scaffold cell repopulation studies. Depending on the scaffold type, surface seeded cells may interact with predominantly BM (eg, luminal surface of vascular, endo or epicardial surface of cardiac muscle, mesothelial surface of viscera, or mesothelial surface of pericardial scaffolds) or type I collagen (eg, mediastinal surface of pericardium, epimysium of skeletal muscle, or abluminal surface of vascular scaffolds) containing ECM niches. As previously discussed, BM components have been shown to represent a potent barrier to Z-direction migration of both endothelial and mesothelial cells.^16,54,55,59,98^ Such findings are consistent with the known physiologic functions of BM components such as Laminin α5 which is known to inhibit leukocyte transmigration across the vessel wall.^99^ Although Z-direction migration is improved following surface seeding on non-BM surfaces, most studies demonstrate that the majority of seeded cells still remain on the scaffold surface.^16,59,71^ Recent studies have shown that decellularization/antigen removal approaches which retain native tissue ECM scaffold molecular cue are capable of increasing the proportion of seeded cells that penetrate in the Z-direction by up to 70%.^10^ However, even in such scaffolds, the proportion of parenchymal void spaces filled with seeded cells remains low. This finding is likely due to the limitation of total number of cells that can be seeded before confluence at the surface is reached. Consequently, for surface seeding to be successful in repopulating a higher proportion of parenchymal void spaces, seeding of BM-containing niches may need to be avoided and either long enough culture durations to allow in situ expansion of cells that achieve Z-direction migration or multiple seeding rounds to facilitate multiple opportunities for Z-direction migration to occur are likely to be needed.

### Injection of Cells into the ECM Scaffold

Injection of cells directly into ECM scaffolds attempts to overcome the limitations of surface seeding by delivering cells directly to the ECM niche of interest.^33,100,101^ Ott et al decellularized rat hearts by coronary perfusion with detergents, preserved the underlying extracellular matrix, and produced an acellular, perfusable vascular architecture, competent acellular valves, and intact chamber geometry.^33^ Perfusion decellularized scaffolds were reseeded with intramural injection of neonatal cardiac cells and perfusion of rat aortic endothelial cells into the vascular conduits. The resulting heart constructs showed contractile responses to electrical pacing stimuli and were responsive to phenylephrine drug administration on day 8 of culture. Recellularization was greatest (33.8 ± 3.4% recellularization) in the area of injection (ie, left ventricular mid-wall). By day 8, there were areas of confluent cellularity approximately 1 mm thick and viability was >95% throughout the entire thickness (0.5-1.1 mm). However, recellularization rapidly diminished at remote sites distant from the injection (ie, 1-2 mm). Although this landmark publication demonstrated the potential benefits of perfusion-based decellularization, the extent to which injection-seeded primary cells migrated into and oriented with the parenchymal void spaces remains unclear. Additionally, since multiple different cell types were delivered in each injection (ie, neonatal cardiomyocytes, fibroblasts, endothelial cells, and smooth muscle cells) the influence of each cell type in achieving the observed confluent cellularity is difficult to assess. Shirakigawa et al compared the efficiency of void space repopulation following recellularization of decellularized rat livers with primary rat hepatocytes delivered via parenchymal injection, hepatic view perfusion, or portal vein perfusion. The authors found that hepatocyte perfusion resulted in clogging of the vasculature with primary hepatocytes, with minimal migration of the cells into the parenchymal spaces. Parenchymal injection resulted in significantly greater void space repopulation and enhanced hepatocyte function (ie, albumin production). However, as seen in other injection seeding publications, void space repopulation was largely confined to the site of injection. The above results demonstrate that it is difficult to know if the cells simply proliferate in situ at the injected site, without truly integrating with the scaffold, or migrate into the ECM void spaces and fully integrate with the scaffold following injection seeding. Consequently, future studies are required to clearly define the migratory potential of various injected cell types, characterize the extent of cellular integration within the ECM (eg, individual cells surrounded by scaffold ECM), and determine the capacity of the ECM to modulate the orientation of seeded cells within the parenchymal void spaces. Even if injection-seeded cells are able to migrate and integrate with the ECM void spaces of the scaffold, delivery of a large enough number of cells via this approach to repopulating all void spaces remains challenging.

### Perfusion Recellularization of Parenchymal Void Spaces

**Figure 3.**
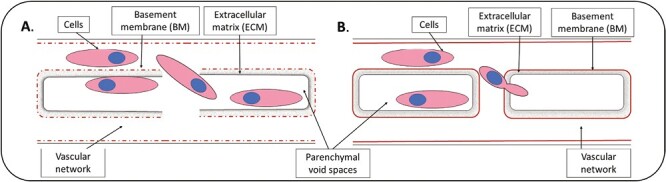
Cell repopulation of parenchymal void spaces following perfusion recellularization depends on vascular tree integrity. **A**. Damage to the vascular tree during decellularization leaves communications between the vascular tree and parenchymal void spaces, results in potential for perfusion-based parenchymal void space repopulation to be falsely achieved. **B**. Intact vascular tree following decellularization requires perfused cells to extravasate and migrate through the interstitial ECM to achieve void space repopulation.

Perfusion recellularization has the advantage of facilitating the delivery of cells to within the diffusional distance of all parenchymal void spaces of the tissue. Indeed, depending on the organ and perfusion route delivery of parenchymal cells directly to all parenchymal void spaces may be achieved without the need for migration of the delivered cells (eg, lung parenchyma seeding via airway perfusion).^70,102-104^ However, as discussed in the vascularization section, perfusion-based seeding approaches are critically dependent on preservation of native tissue ECM integrity and associated niche properties. Disruption of vascular beds during scaffold preparation may allow perfused cells to leak into parenchymal void spaces, creating the false appearance of void space repopulation (Fig. 3). Consequently, careful evaluation of ECM scaffold integrity prior to perfusion seeding is essential to accurately interpret reseeding results. In the case of solid organ engineering, vascular perfusion with cells of endothelial lineage has the potential to result in the repopulation of intact vascular beds. However, as previously discussed, the extent to which intact vascular BM’s serve as a barrier to Z-direction migration of cells into the remaining parenchymal void spaces remains to be determined.^59^ Consequently, although retention of the native vascular tree is essential for recapitulation of a perfusable vasculature, the ability of BMs to prevent Z-direction migration presents a competing challenge for perfusion-based recellularization of the parenchymal void spaces.

### Perfusion Recellularization of Hollow Organs

In the case of lung perfusion recellularization, vascular perfusion has been used to deliver endothelial cells to the vascular bed, while airway perfusion has been used to deliver epithelial cells to the respiratory tree.^69^ Petersen et al used intratracheal perfusion-based seeding of a mixed population of neonatal rat lung epithelial cells, resulting in cellular repopulation of alveolar structures, as well as small and medium-sized airways.^95^ Microvascular lung endothelial cells, were also injected into the pulmonary artery of acellular scaffolds which adhered throughout the scaffold vasculature. Similarly, Ott et al studied the repopulation of the lung with HUVECs (via vascular perfusion) and carcinomatous human alveolar basal epithelial cells (cell line A549) via transtracheal perfusion.^91^ This approach led to dispersion of endothelial cells throughout the entire pulmonary vascular tree. Simultaneously, epithelial cells showed engraftment and formation of an epithelial monolayer after 5 days in culture throughout the entire lung construct, with preservation of parenchymal architecture. Consequently, perfusion-based seeding approaches show clear promise for increasing the number and distribution of delivered cells in ECM scaffold void spaces, although current progress in solid organs lags behind that of organs containing multiple potential perfusion routes (eg, lung and liver).

### Perfusion Recellularization of Solid Organs

For solid organs, assuming perfect maintenance of ECM structure throughout the decellularization process, no perfusion route exists which directly communicates with the parenchymal void spaces. Consequently, repopulating cells are required to migrate from the perfusable vascular tree (ie, across the vascular BM), through the interstitial ECM (predominantly type I collagen in the majority of organs), and ultimately into the parenchymal void spaces (Fig. 3). To achieve such migration, repopulating cells are required to either change their morphology to fit through pores in the ECM, or enzymatically digest some of the matrix to facilitate cellular migration. The amount of matrix remodeling undertaken by the cells depends on the ability of the cell to digest matrix, and the matrix structure; in “looser” matrices, cells can migrate freely without requiring proteolysis, but in a less porous structure more digestion may be required.^105,106^ Furthermore, compaction of the ECM may occur due to loss of primary cell pre-stress on the ECM following decellularization further compromising seeded cell migration. Furthermore, specialized ECM proteins (eg, laminin α5) are known to serve as barriers to cell migration (ie, translocation across vascular BMs to achieve Z-direction migration into parenchymal void spaces) and must therefore be considered in attempts to drive void space migration and repopulation.^99^ Consequently, ECM porosity, composition (eg, specific niche proteins such as BM components), cell migration capacity, and cellular function (eg, ability to remodel ECM to facilitate migration) should all be taken into account when designing solid-organ perfusion recellularization approaches.

Given the challenges inherent to achieving Z-direction migration of perfusion-seeded cells across the vascular BM, through the interstitial ECM and into void spaces, it is unlikely that full repopulation can be achieved without providing some external factors to influence the proportion, direction, and speed of cell migration through ECM scaffolds. Consequently, a number of groups have recently focused on determining the mechanisms by which in vitro XY (ie, across planar surface) and Z direction cell migration throughout ECM scaffolds can be controlled. Signals for cell migration maybe soluble (eg, chemokines, cytokines, or growth factors), matrix bound (eg, matrikines, matricryptic signals, matrix bound vesicles, and growth factors), or mechanical (eg matrix rigidity and physiologically applied static or cyclic stresses).^107^

### Methods to Control Cell Migration

In the case of soluble signals, the most commonly used growth factors (GFs) have included bone morphogenic proteins (BMP),^108^ basic fibroblast growth factors (bFGF, bFGF-2),^109^ vascular epithelial growth factor,^110^ and transforming growth factor-beta (TGF-β).^111^ GFs control a wide range of biological functions, including cell proliferation, survival, migration, and differentiation. For example, Leuning et al bioengineered re-endothelialized whole kidney matrix scaffolds from both rodents and humans using human inducible pluripotent stem cell-derived endothelial cells along with vascular endothelial growth factor and angiopoietin 1 while achieving efficient cell delivery, adherence, endothelial cell proliferation, and survival.^67^ The processes of cell migration, differentiation, and proliferation are typically dependent on both the presence of specific growth factors and their spatial- and time-dependent distributions. Consequently, the most critical issue related to growth factors is delivery of physiologically relevant doses and control of the duration of action of such molecules at the site of action due to their burst release kinetics and localized concentration following injection-based delivery strategies.^112^ To overcome the deficiencies of direct injection, several ways of GFs delivery have been developed such as cell transfection with DNA plasmids encoded with the desired GF, introducing an inducible GF gene into the cellular genome (gene editing) and implantation of GF proteins directly into the cell.^113,114^

Chemotactic gradients can be applied to encourage cell migration across scaffolds, employing a variety of matrix materials as well as cell types.^115-118^ Although these studies have demonstrated successful control of cell migration, they have predominantly used hydrogel-based scaffolds, while controlled recellularization of decellularized ECM scaffolds through chemotactic gradients has not been widely reported. Furthermore, as previously discussed, poor void space filling deep within tissues presents a challenge; directing migration into tissues (Z-direction) is much more difficult than migration across scaffold surfaces (X- or Y-direction), at least in part because of the fact that BMs present a barrier to Z-direction migration.^59^ Some groups have achieved early success by using external factors to influence Z-direction migration of cells into the matrix. Pre-treating scaffolds with exogenous factors (eg, fibronectin coating) have been used to accelerate initial cell migration into decellularized ECM scaffolds.^119^ Assmann et al demonstrated that fibronectin coating was successful in accelerating autologous in vivo recellularization of aortic conduits following implantation in Wistar rats for up to 8 weeks. Fibronectin coating resulted in significantly accelerated luminal endothelialization (*P* = .006 after 8 weeks), and enhanced myofibroblast hyperplasia, significantly increasing the intima-to-media thickness ratio (*P* = .0002 after 8 weeks). Alpha-smooth muscle actin and desmin positive cell invasion into the vascular media of fibronectin-coated conduits originating from the adventitial surface was significantly increased as compared to uncoated control conduits (*P* < .0001). This work shows that biofunctional protein coating accelerated in vivo endothelialization and induced a significantly increased medial recellularization, however, the effects of such pre-treatment did not last and still resulted in incomplete void space repopulation deeper in the tissue.^119^ Additionally, despite the promise of such ECM coating approaches, dose-dependent control of such stimuli must be achieved to avoid potential negative consequences (eg, intimal and/or medial hyperplasia). Further research into the manipulation of chemotactic gradients in vitro is needed before an optimal combination of seeding method and culture conditions is established. Achieving control of cellular migration in ECM scaffolds could enable the complete repopulation of void spaces, a goal that has evaded tissue engineers for some time. Others have investigated nutrient sources in the perfusing media as a driver of cellular migration.^120^ Tosun et al aimed to enhance cellular migration by applying directed mechanical and nutritional gradients to promote transmural migration and more uniformly populate vascular constructs. Using perfusion bioreactors, lumen pressure (either 120/80 mmHg [high pressure] or 50/30 mmHg [low pressure]) was used to modulate transmural hydraulic conductivity, either with, or without, media in the abluminal flow circuit. Cells seeded on the abluminal surface migrated toward the lumen when media was perfused in the luminal flow path only, regardless of perfusion pressure. However, the extent of migration was maximized when low-pressure perfusion (SP-Lp: 50/30 mmHg) was used compared to the high-pressure perfusion group. Both of the above studies use ­vascular scaffolds, which easily enable separate control of the luminal and abluminal conditions. This method allows easy and effective establishment of chemotactic and/or transmural pressure gradients, but translation of these principles to whole organs may present challenges since isolation of the perfusable lumen from the parenchymal void spaces is more challenging in such geometries.

Cell migration is also influenced by matrix factors such as stiffness and porosity. This is particularly important with scaffolds derived from natural tissues where pore size is subcellular limiting cell seeding and adhesion to the scaffold periphery. The density and spatial alignment of the scaffold determine the mechanical tissue properties (ie, stiffness and porosity), which guide or oppose cell migration and positioning. Wolf et al expressed that the structural characteristics of connective tissue-derived collagen display a dual function for cell movement as (1) guidance structure providing preformed tracks, and (2) barrier functions by forming fiber networks with random gaps of subcellular size.^121^ Recent studies have revealed the importance of biomechanical conditions in addition to biochemical cues for cell signaling and migration.^122^ It has been suggested by Zhao et al, that biochemical cues are better at guiding cell migration with improved directionality and persistence, while mechanical cues are better at coordinating collective cell migration.^123^ Extent to which such reductionist approaches could be applied to manipulate the properties of naturally derived ECM scaffolds remains underexplored and may represent an area for future advances.

## Cell Differentiation

Although it is possible for tissue engineering efforts to employ primary cells in some settings (eg, endothelialization) lack of migratory capacity, limited availability, and/or poor proliferation capacity of many primary cell types (eg, cardiomyocytes) drive the need to employ progenitor and/or stem cells for parenchymal void space seeding in many solid-organ engineering applications.^124^ This necessity drives the need to understand and control in situ differentiation of such stem/progenitor cells following parenchymal void space repopulation. While the previously mentioned challenges of void space repopulation have hindered progress in the ECM scaffold-driven cell differentiation field, some limited successes have been reported. Early attempts to examine the potential for ECM scaffolds to modulate cell differentiation used micronized (eg, lyophilized and pulverized) ECM scaffolds (Table 1C). For instance, Fong et al showed that maturation of human iPSC-derived cardiomyocytes was enhanced following seeding on native micronized cardiac ECM compared to 2D culture, as demonstrated by increase expression of calcium handling genes and associated calcium signaling kinetics.^124^ However, such experiments lack some of the potentially critical signaling factors (eg, ECM orientation and mechanics) which may be capable of driving cell differentiation. Insights from reductionist approaches indicate that many of the signals which are capable of stimulating migration (eg, soluble, matricryptic, and mechanical) are also capable of influencing cell differentiation or maintaining primary cell differentiating. For example, Kudva et al showed that primary human articular chondrocytes (hAC) de-differentiate when grown on 2D surfaces, but 3D polyethylene glycol (PEG) hydrogels incorporated with RGD maintained hAC viability and chondrogenic gene expression over time.^125^ Similarly, Flaim et al explored the feasibility of using ECM protein microarrays to study both primary rat hepatocyte dedifferentiation and embryonic stem (ES) differentiation toward early hepatic lineage. Combinations of ECM proteins were identified that were capable of driving ES differentiation and preventing de-differentiation of primary cells.^126^ Consequently, there is evidence that ECM components are capable of driving stem cells and maintaining terminal cell differentiation. The development of perfusion decellularization and recellularization approaches facilitated the study of site-specific (eg, vessel vs. parenchymal void space) differentiation potential of intact ECM to be studied.

Due to the limited supply of autologous primary cells and their phenotypic instability following in vitro proliferation, stem cell plasticity in tissue culture has been widely explored. For this purpose, multi- or pluripotent stem cells were isolated from various regions of the body, ranging from tissue-specific progenitor cells, to circulating progenitors and mesenchymal stem cells (MSC).^127^ MSCs have received particular attention due to their tri-lineage differentiation potential and immunomodulatory properties, which may have benefits in modulating residual ECM scaffold-specific immune responses.^128^ However, the multipotent potential of MSCs proved to be a major limitation to their use in whole organ engineering efforts, since they were incapable of differentiation to many of the required primary parenchymal cell types. Unlike MSCs, human embryonic stem cells (hESCs) derived from the inner core of blastocytes have pluripotent capacity. This benefit has led to use of hESCs in a number of recent publications.^129^ For example, Rajabi et a generated decellularized rat hearts, with or without immobilized basic fibroblast growth factor (bFGF), followed by perfusion reseeding with human embryonic stem cells (hESCs)-derived cardiovascular progenitor cells (CPCs). bFGF immobilization improved retention of CPCs, with cells demonstrating ECM site-specific differentiation toward cardiomyocyte, smooth muscle cell, and endothelial cell lineages.^72^ However, although parenchymal void space repopulation reached 50.2% in the LV free wall, the remainder of the myocardial tissue was less well populated (~24% void space filling). Despite the current technical limitations of parenchymal void space repopulation, such studies demonstrate the potential of ECM scaffolds to inform site-specific cell differentiation.

Development of induced pluripotent stem cells (iPSC) has further fueled interest in in vitro ECM scaffold cellular repopulation and differentiation studies.^130^ Alexanian et al used perfusion-based seeding of decellularized mouse heart ECM scaffolds with induced cardiac progenitor cells (iCPC) reprogrammed from either lung or cardiac fibroblasts.^74^ Following seeding the ECM scaffold drove iCPCs differentiation toward cardiomyocytes, endothelial, and smooth muscle lineages in a site-specific manner (ie, endothelial and smooth muscle cell differentiation in vascular locations, cardiomyocyte differentiation in parenchymal void space locations).^74^ The differentiated cardiomyocytes showed an increase in cell size and sarcomere organization compared to in vitro differentiated iCPCs, suggesting that in addition to the growth factors and small molecules that were perfused to promote differentiation of iCPCs, the appropriate cardiac ECM niche further facilitated differentiation. Despite these promising results, the proportion of cardiomyocyte to endothelial cell differentiation was far from that expected for native cardiac muscle. Similarly, Hochman-Mendez et al used sequential perfusion seeding with human iPSC-derived endothelial cells and cardiomyocytes to repopulate decellularized rabbit whole heart ECM scaffolds.^49^ Seeded cells regenerated left ventricular (LV) wall to full thickness from base to apex and endocardium to epicardium. However, cellular repopulation of the interventricular septum and right ventricular free wall was less successful. Although cardiac and endothelial cell site-specific differentiation was observed, as with other similar studies the ratio of endothelial cells to cardiomyocytes was far lower than expected for native cardiac muscle. Consequently, although this study demonstrated the potential for ECM to inform iCPC cell differentiation in a site-specific manner, future work is needed to further understand and optimize the ECM factors responsible for driving such differentiation.

Functionalization of ECM scaffolds to further enhance cell function/differentiation has also been studied. For example, Shaohua Ge et al fabricated a nanostructured hydroxyapatite-coated porcine acellular dermal matrix (HAp-PADM) by a biomimetic mineralization method and seeded human periodontal ligament stem cells (PDLSCs).^131^ The effects of this scaffold on cell shape, cytoskeleton organization, cell viability, and osteogenic differentiation were examined. The results indicated that periodontal ligament stem cells cultured on HAp-PADM exhibited different cell shapes when compared with those on pure PADM. Moreover, HAp-PADM promoted cell viability and increased alkaline phosphatase activity significantly. It was concluded that the surface HAp nanonetwork structure of PADM could promote the viability and differentiation of PDLSCs and is a promising scaffold for periodontal tissue engineering.^131^ However as for non-functionalized scaffolds this field is hindered by the incomplete void space repopulation issue which results in cells that may or may not be interacting with native ECM across their entire surface.

Consequently, although current studies show that the ECM has potential to drive cell differentiation beyond embryonic stages, several barriers exist which currently prevent the full potential of this field from being realized. Solving the parenchymal void space repopulation issue is the first critical barrier to progress since without ensuring correct orientation of repopulating cells within the 3-dimensional matrix the signals they receive will at best be “diluted” (eg, cells sitting on the scaffold surface receive signals from only that contact surface and additionally may not be exposed to the full complement of matrix-bound signals deeper in the ECM). Even if the void space issue can be solved, future work focusing on the effect of scaffold preparation techniques on ECM-bound growth factors, matricryptic signal, matrikines, and mechanical properties and their effects on cell migration, proliferation, function, and differentiation will be required before the full potential of ECM scaffolds can be harnessed.

## Data Availability

No new data were generated or analyzed in support of this research.
